# Vitexin induces apoptosis by suppressing autophagy in multi-drug resistant colorectal cancer cells

**DOI:** 10.18632/oncotarget.22890

**Published:** 2017-12-04

**Authors:** Monika Bhardwaj, Hee Jun Cho, Souren Paul, Rekha Jakhar, Imran Khan, Seon-Jin Lee, Bo-Yeon Kim, Manigandan Krishnan, Tejinder Pal Khaket, Hee Gu Lee, Sun Chul Kang

**Affiliations:** ^1^ Department of Biotechnology, Daegu University, Kyoungsan, Kyoungbook, Republic of Korea; ^2^ Immunotherapy Convergence Research Center, Korea Research Institute of Bioscience and Biotechnology, Daejeon, Republic of Korea; ^3^ Department of Biomolecular Science, University of Science and Technology (UST), Daejeon, Republic of Korea; ^4^ World Class Institute, Anticancer Agents Research Center, Korea Research Institute of Bioscience and Biotechnology, Ochang, Cheongwon, Republic of Korea

**Keywords:** apoptosis, autophagy, colorectal cancer, multidrug resistance, vitexin

## Abstract

Cancer treatment is limited due to the diverse multidrug resistance acquired by cancer cells and the collateral damage caused to adjacent normal cells by chemotherapy. The flavonoid compound vitexin exhibits anti-oxidative, anti-inflammatory and anti-tumor activity. This study elucidated the antitumor effects of vitexin and its underlying mechanisms in a multi-drug resistant human colon cancer cell line (HCT-116^DR^), which exhibits higher levels of multidrug-resistant protein 1 (MDR1) expression as compared with its parental cell line (HCT-116). Here, we observed that vitexin suppressed MDR-1 expression and activity in HCT-116^DR^ cells and showed cytotoxic effect in HCT-116^DR^ cells by inhibiting autophagy and inducing apoptosis in a concentration-dependent manner. Additionally, vitexin treatment caused cleavage of caspase-9 and caspase-3, and upregulated the expression of the pro-apoptotic proteins, BID and Bax. Moreover, the expression of autophagy-related proteins, such as ATG5, Beclin-1 and LC3-II, was markedly reduced by vitexin treatment. Furthermore, *in vivo* experiments showed that vitexin induced apoptosis and suppressed tumor growth in HCT-116^DR^ xenograft model. These results revealed that vitexin induced apoptosis through suppression of autophagy *in vitro* and *in vivo* and provide insight into the therapeutic potential of vitexin for the treatment of chemo-resistant colorectal cancer.

## INTRODUCTION

Colon cancer is the third most diagnosed cancer and the leading cause of cancer-related mortalities worldwide. Colon cancer is treated by chemotherapy or surgery depending upon cancer stage, tumor location at diagnosis and individual characteristics of the patients [1]. Despite previous research efforts, some colon cancer patients fail to respond to conventional treatment due to resistance acquired by cancer cells against structurally and mechanistically unrelated compounds known as multi drug resistance (MDR), which causes recurrent tumor progression [2]. Currently, most research is streamlined for the development of natural anticancer agents that possess low toxicity and overcome MDR phenotypes to decrease limitations associated with the cure rate and probable survival of cancer patients. One of the mechanisms of MDR is associated with the active extrusion of antineoplastic agents from cancer cells through transporter proteins, which reduces the intracellular concentration of the drug below the cytotoxic threshold [3]. Drug efflux from cancer cells is tightly regulated by ATP binding cassette (ABC) transporter family proteins. High level expression of ABC proteins is observed in various human cancers and results in development of resistance to multiple anti-cancer drugs [4].

The P-glycoprotein (P-gp), also known as MDR1 or ABC sub-family B member 1 (ABCB1), is a well characterized plasma-membrane protein that pumps many foreign substances out of cells. This protein is upregulated in several cancers and responsible for decreased drug accumulation in MDR cells, thereby promoting development of resistance to anti-cancer drugs [5]. Therefore, inhibition of P-expression or function is proposed as effective approaches sensitizing drug-resistant cancer cells to chemotherapeutic agents, however, most results obtained from *in vivo* studies show that modulators of P-gp activity are ineffective [6]. Therefore, P-gp inhibitors derived from natural products and their synthetic derivatives are considered as potential candidates to reverse the effects of drug resistance in human cancer cells.

Accumulating evidences indicates that altered-expression of anti-apoptotic or pro-apoptotic proteins could lead to drug resistance against chemotherapy. Macroautophagy (autophagy) is a cellular degradation pathway that eliminates damaged organelles or unused proteins and is involved in facilitating colon cancer progression and promoting resistance against stress. Cancer cells utilize autophagy, typically activated under metabolic stress or amino acid starvation condition, to support the survival and growth of established tumors [7]. Previous studies suggested that autophagy promotes cancer cell resistance against chemotherapy treatment by inhibiting apoptosis, and that autophagy inhibition potentiates resensitization of therapeutic resistant cancer cells to chemotherapy [8, 9]. Therefore, targeting the autophagic process using several pharmaceutical agents might prove beneficial to combating drug resistance of cancer cells.

Vitexin is a naturally derived flavonoids compound that exhibits anti-oxidative, anti-inflammatory and analgesic activities. This compound has also shown anti-tumor efficacy against various human cancers, including ovarian, esophageal, prostate, and breast cancers, which involve its capacity to promote apoptosis of cancer cells [10, 11]. However, it remains unclear whether vitexin also induces apoptosis in chemo-resistant cancer cells. In this study, we investigated the cytotoxic effect of vitexin against drug-resistant cancer cells. To this end, we developed an MDR human colorectal carcinoma cell line, HCT-116^DR^, established by exposuring the parental colorectal cancer HCT-116 cell line to 5-fluorouracil, cisplatin, docetaxel and vincristine. Our results indicated that vitexin treatment induced apoptosis by suppressing autophagy in HCT-116^DR^ cells. These findings provide mechanistic insight into the cytotoxic effects of vitexin in MDR human colorectal cancer cells.

## RESULTS

### The effects of vitexin on the viability of the drug-resistant HCT-116^DR^ cell line

**Figure 1:**
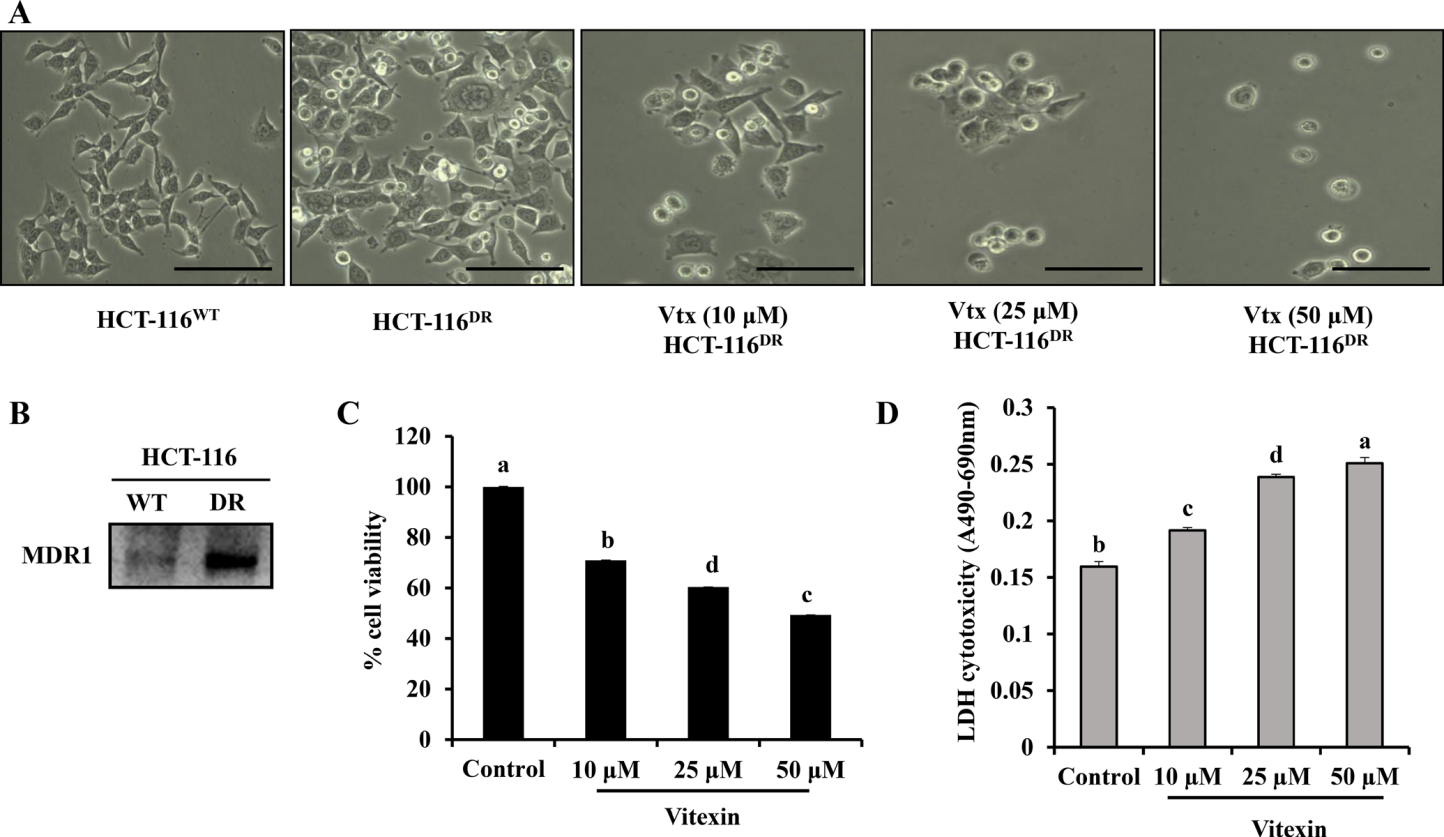
Growth inhibition of HCT-116^DR^ cells by vitexin treatment (**A**) Cell morphological changes after treatment with 10 μM, 25 μM and 50 μM vitexin for 24 h. (**B**) Expression of MDR1 in HCT-116 and HCT-116^DR^ cells. (**C, D**) Cells were treated with the indicated concentrations of vitexin for 24 h, and cell viability were determined by (C) MTT and (D) LDH assays as described in the Materials and Methods. Data represent the mean ± SD of three independent experiments (*n* = 3). Values with different letters (a–d) denote significant difference from one another (*p* < 0.05).

To examine the cytotoxic effect of vitexin in chemo-resistant cancer cells, HCT-116^DR^ cells were treated with vitexin at increasing concentrations for 24 h. Vitexin treatment caused morphological changes in HCT-116^DR^ cells, including dose-dependent rounding and detachment from cell culture plates (Figure 1A). Because MDR1 levels are often upregulated in chemo-resistant cancer cells [12, 13], we verified MDR1 expression in parental HCT-116 colorectal cancer cells and HCT-116^DR^ cells. Western blot analysis showed enhanced MDR1 expression in HCT-116^DR^ cells as compared with levels observed in parental HCT-116 cells (Figure 1B). To investigate the effects of vitexin on cell viability, we performed 3-(4,5-dimethylthiazol-2-Yl)-2,5-diphenyltetrazolium bromide (MTT) assays. We observed that treatment of HCT-116^DR^ cells with vitexin significantly decreased cell viability in a dose-dependent manner (Figure 1C). Furthermore, vitexin-treated cells exhibited increased lactate dehydrogenase (LDH) activity (Figure 1D), suggesting that vitexin inhibited the viability of drug-resistant colorectal cancer cells. In addition, the cytotoxic effect of vitexin in different cell lines was also checked by MTT assay. Vitexin (5–100 µM) was treated for 24 h in colon, lung, liver and cervical cancer cell lines. We observed that vitexin markedly reduced the cell viability in these cell lines (Supplementary Figure 1), which suggest that vitexin can exert its cytotoxicity against diverse range of cell lines.

### Vitexin suppresses MDR1 expression and activity

**Figure 2:**
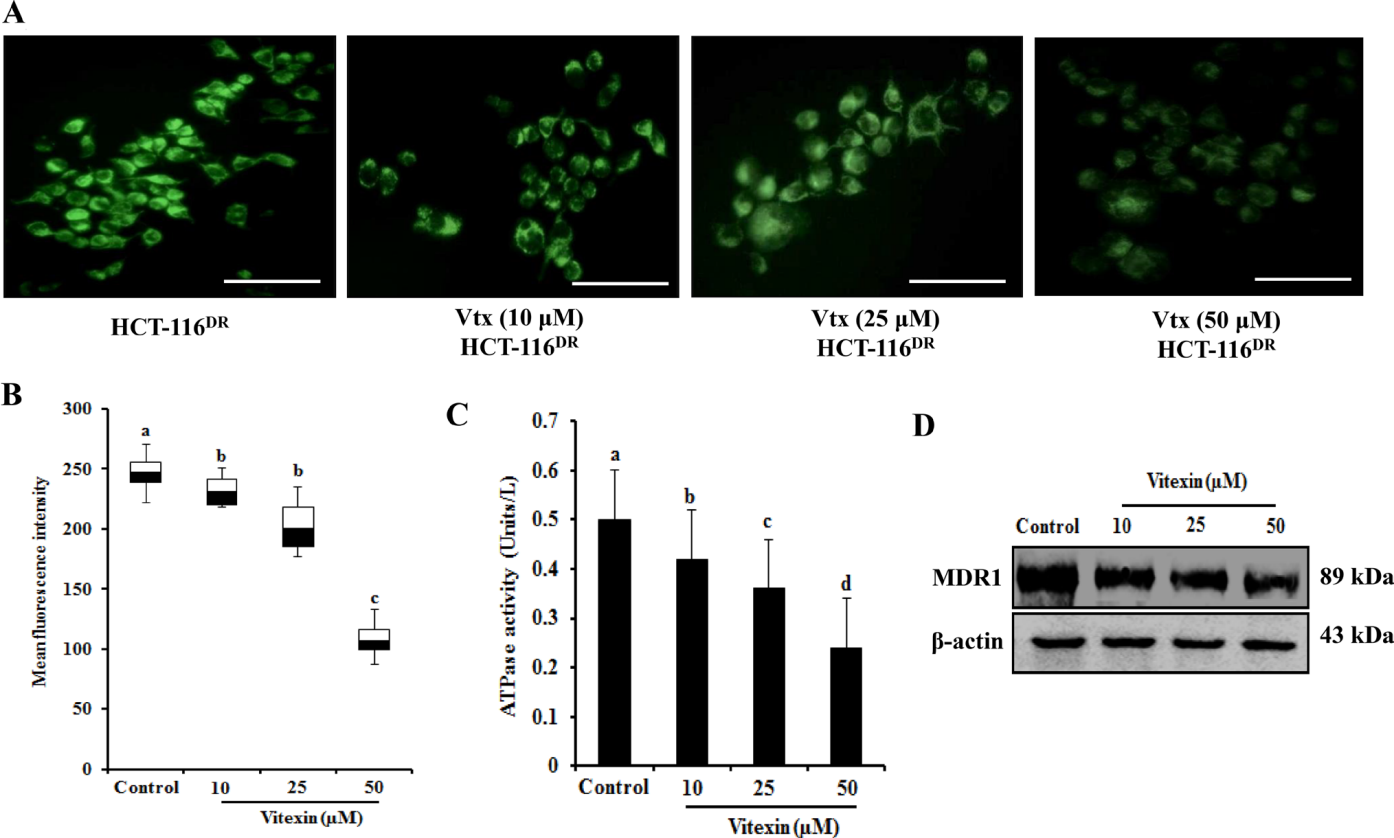
Vitexin diminishes Rh-123 accumulation (**A, B**) Effects of vitexin treatment on the Rh-123 accumulation in HCT-116^DR^ cells. Cells were pre-incubated in the presence or absence of vitexin for 24 h at 37°C, followed by incubation with Rh-123 for another 30 mins at 37°C. Accumulation of Rh-123 was measured by fluorescence microscopy. (**C**) Effects of vitexin treatment on ATPase activity in HCT-116^DR^ cells. (**D**) Cells were treated with vitexin at the indicated concentrations, and total cell lysates were immunoblotted with the MDR1 antibody. Data represent the mean ± SD of three independent experiments (*n* = 3). Values with different letters (a-d) denote significant difference from one another (*p* < 0.05).

Because MDR-1 expression is upregulated in HCT-116^DR^ cells, we investigated whether vitexin treatment affects MDR1 expression and/or activity. Intracellular accumulation of rhodamine-123 (Rh-123) was measured to determine MDR1 activity in HCT-116 ^DR^ cells treated with various concentrations of vitexin for 24 h. As shown in Figure 2A and 2B, vitexin-treated HCT-116^DR^ cells showed Rh-123 accumulation relative to control cells, as well as marked reductions in MDR1-related ATPase activity in a dose-dependent manner (Figure 2C). We then assessed whether vitexin could affect MDR-1 protein levels. HCT-116^DR^ cells were treated with vitexin at increasing concentrations for 24 h, and cell lysates were subjected to western blot analysis. Our results showed significantly reduced MDR-1 levels following vitexin treatment (Figure 2D). These data indicated that vitexin suppressed MDR1 expression and subsequently attenuated its activity.

### Effects of vitexin on apoptosis induction in HCT-116 ^DR^ cells

**Figure 3:**
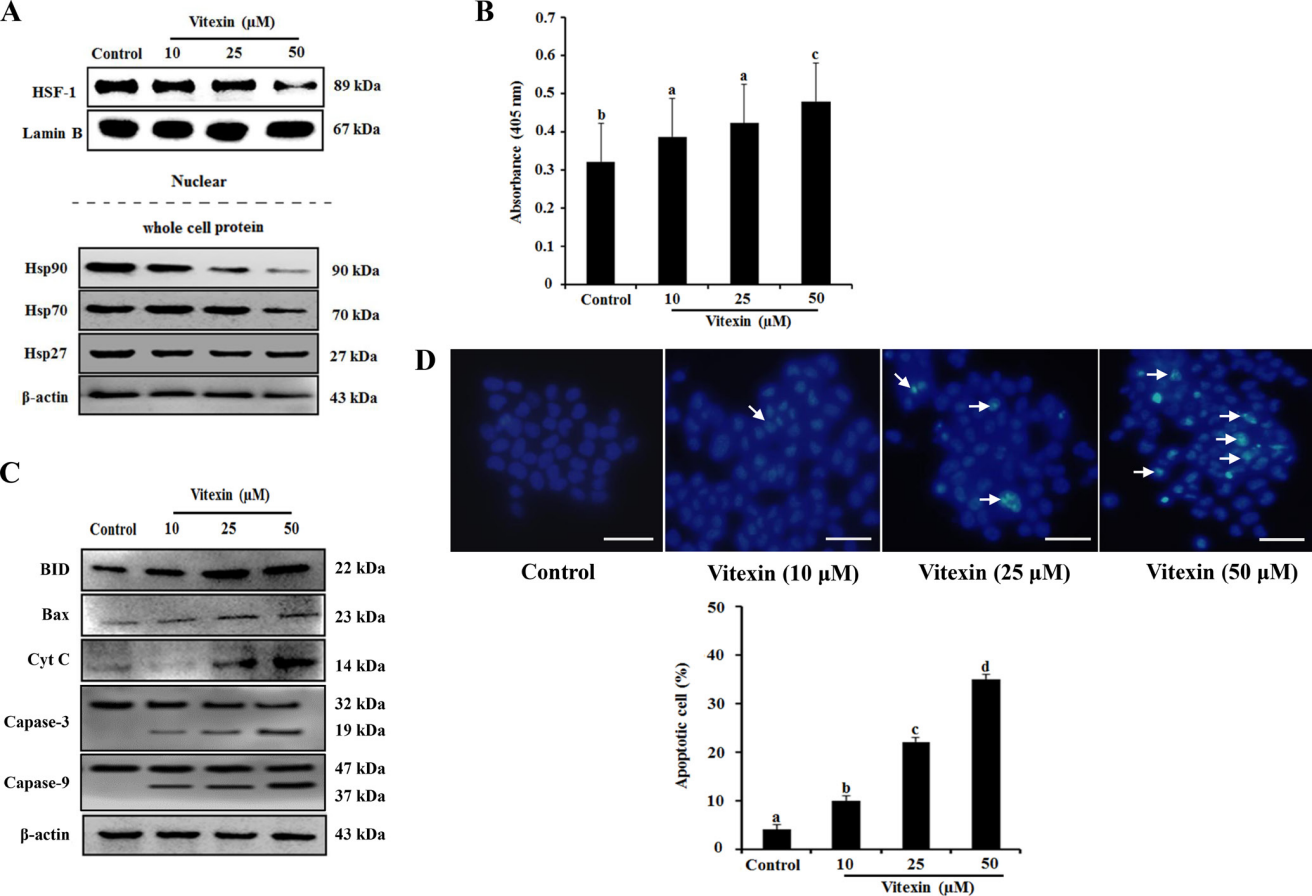
Vitexin treatment abolishes nuclear translocation of HSF-1 and induces apoptosis HCT-116^DR^ cells were treated with vitexin at the indicated concentrations for 24 h. (**A**) Nuclear extracts and total cell lysates were immunoblotted with the indicated antibodies. (**B**) Results of Apostrand ELISA assays following treatment of HCT-116^DR^ cells with vitexin for 24 h. (**C**) Apoptosis-marker proteins were detected by western blotting following treatment of HCT-116^DR^ cells with vitexin for 24 h. (**D**) HCT-116^DR^ cells were stained with Hoechst 33342, after which cell nuclei were observed under a microscope to detect apoptosis. The number of apoptotic cells (strong blue staining) increased significantly in a dose-dependent manner as compared with that observed in the control group. Data represent the mean ± SD of three independent experiments (*n* = 3). Values with different letters (a–d) denote significant differences from one another (*p* < 0.05).

Several studies suggested that heat shock transcription factor (HSF)-1 translocates from the cytosol to the nucleus and regulates the expression of heat-shock proteins (HSPs) during proteotoxic stress, thereby resulting in cancer-cell survival and enhanced chemoresistance [14, 15]. Therefore, we investigated whether vitexin affects the translocation of HSF-1 and the expression of HSPs. HCT-116^DR^ cells were treated with increasing concentration of vitexin for 24 h, and nuclear extracts were subjected to western blot analysis. Our results showed that HSF-1 was amply detected in nuclear extract from untreated HCT-116^DR^ cells. In contrast, these levels were remarkably reduced in HCT-116^DR^ cells treated with vitexin (Figure 3A). In agreement with this result, levels of HSP90 and HSP70 proteins were also decreased by vitexin treatment (Figure 3A). We then investigated whether vitexin-induced cytotoxicity was implicated in the induction of apoptosis. HCT-116^DR^ cells were treated with vitexin for 24h, and apoptotic induction was assessed using the ApoStrand apoptosis detection kit. The results showed that vitexin treatment enhanced DNA single-strand breaks (SSBs), which is a typical feature of apoptotic cells (Figure 3B). Furthermore, we detected elevated expression levels of pro-apoptotic proteins, such as BH3-interacting-domain death agonist (BID), B-cell lymphoma (Bcl)-2-associated X protein (BAX) and cytochrome c, and the cleavage of caspase-3 (Figure 3C). Induction of apoptosis by vitexin in HCT-116^DR^ cells was also confirmed by Hoechst 33342 staining with vitexin-treated HCT-116^DR^ cells showing increased pycnonuclei formation, also indicator of apoptosis (Figure 3D). To determine whether vitexin induces DNA double-strand breaks (DSBs), we performed comet assays (single cell gel electrophoresis), finding vitexin-treated HCT-116^DR^ cells showed increased intensity of the comet tail relative to that of the head, reflecting the number of DSBs (Supplementary Figure 2), as compared with control cells. The apoptotic effect of vitexin was further confirmed by terminal deoxynucleotidyl transferase dUTP nick-end labeling (TUNEL) staining and flow cytometric analysis, with gradual increases TUNEL positive (apoptotic) cells observed following vitexin treatment (Supplementary Figure 3). These results suggested that vitexin induced apoptosis in chemo-resistant HCT-116^DR^ cells.

### Vitexin induces apoptosis by inhibiting autophagy

**Figure 4:**
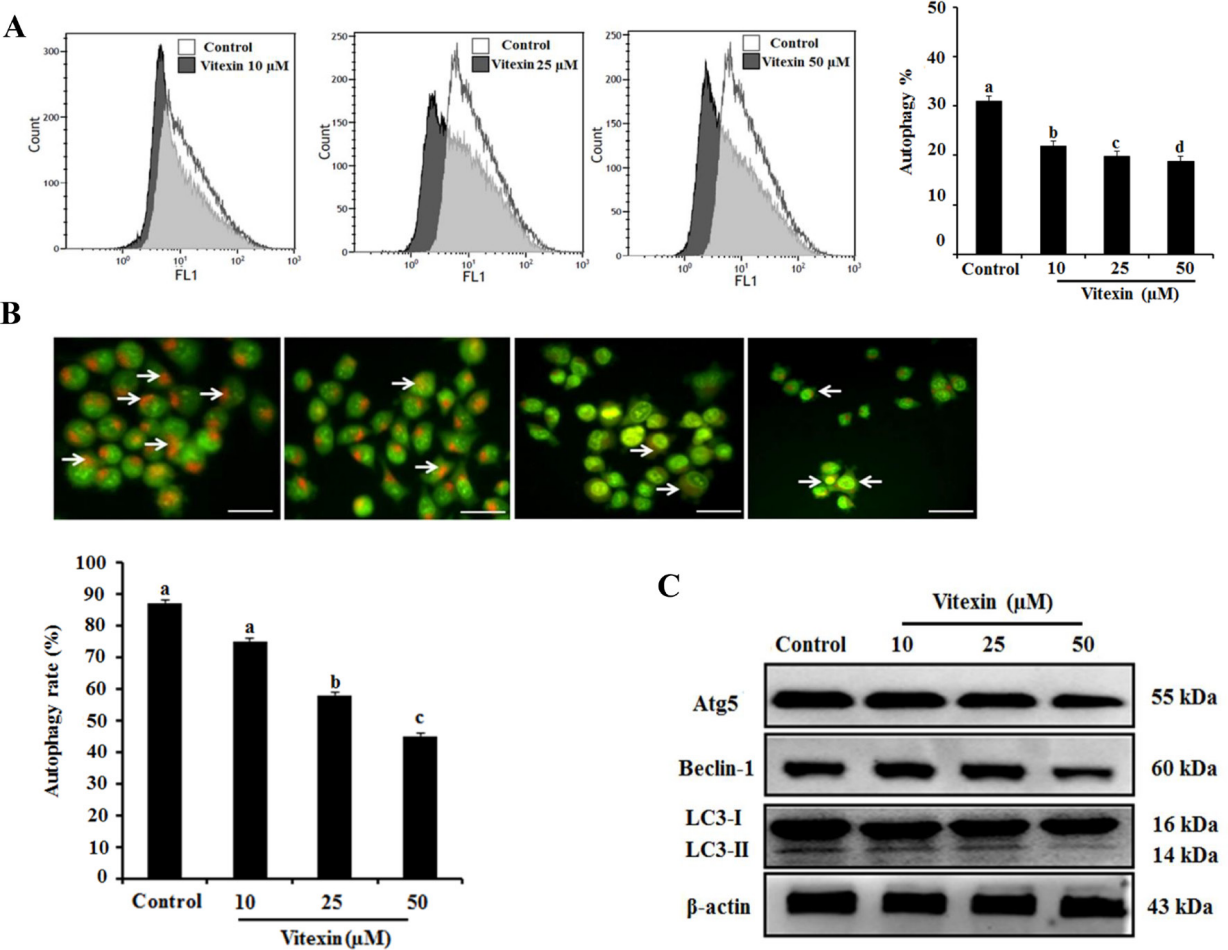
Effects of vitexin treatment on autophagy inhibition in HCT-116^DR^ cells (**A**) Flow cytometric analysis was performed to measure cell autophagy using the Cyto-ID green autophagy reagent. Representative graph of autophagy percentage was determined by flow cytometry following treatment with vitexin for 24 h. (**B**) HCT-116^DR^ cells were subjected to AO staining to allow determination of autophagosome formation (bright orange granules) using a fluorescence microscope (scale bar = 0.1 mm). The percentage of autophagic cells was calculated by observing 400 cells. (**C**) The protein levels of various autophagy markers were analyzed by western blot. Data represent the mean ± SD of three independent experiments (*n* = 3). Values with different letters (a–d) denote significant differences from one another (*p* < 0.05).

**Figure 5:**
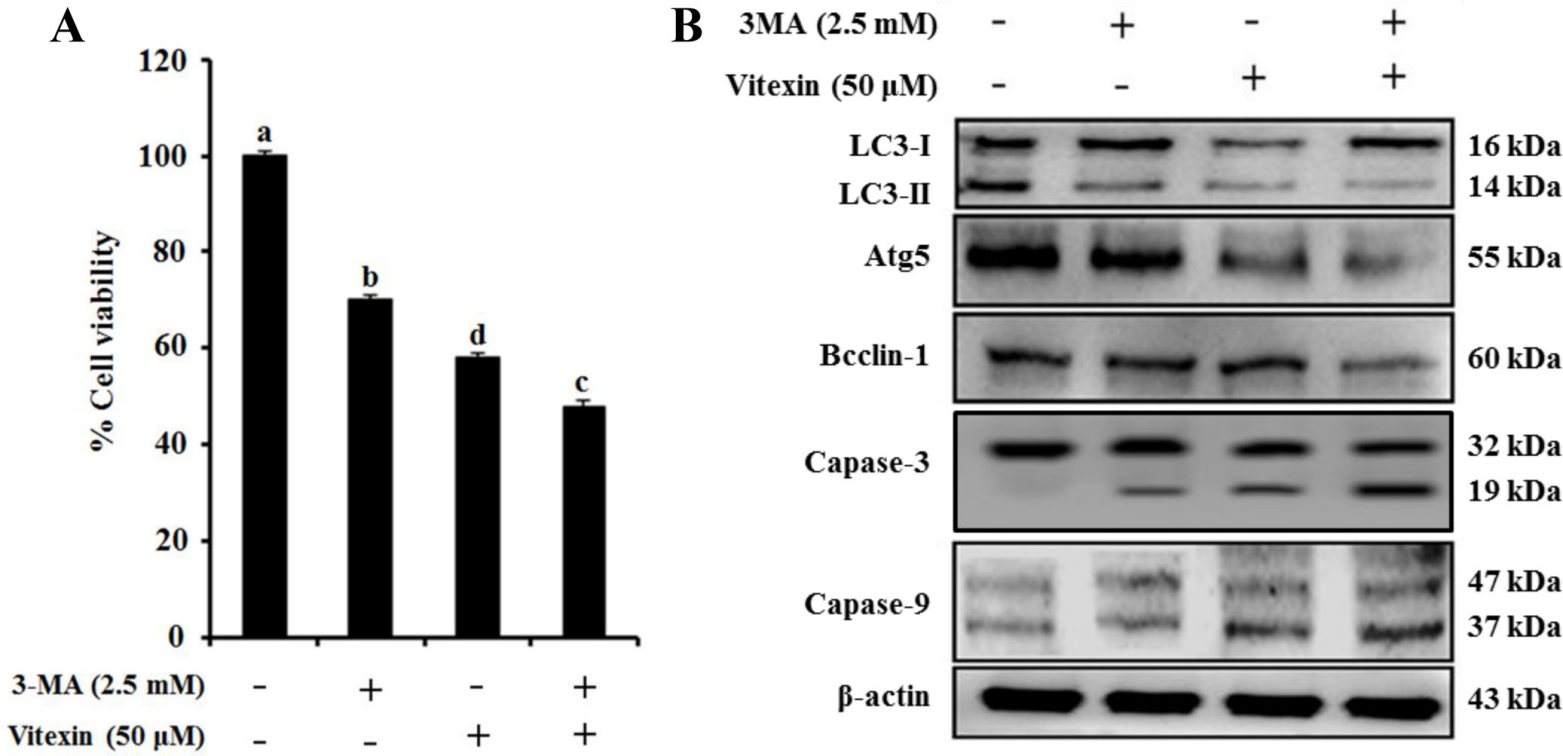
Vitexin treatment reverses the drug resistance of HCT-116^DR^ cells by reducing autophagy (**A**) Cells were treated with 2.5 mM 3-MA and/or 50 μM vitexin for 24 h and effects of 3-MA-mediated autophagy inhibition on vitexin induced cell death were detected by MTT assay. (**B**) Protein levels of various autophagy and apoptosis markers were analyzed by western blot following treatment with 2.5 mM 3-MA and/or 50 μM vitexin for 24 h. Data represent the mean ± SD of three independent experiments (*n* = 3). Values with different letters (a–d) denote significant differences from one another (*p* < 0.05).

Several studies indicate that autophagy promotes cell survival or apoptosis in tumor cells treated with chemotherapeutic agents [16, 17]. Here, we assessed whether vitexin affect autophagy in HCT-116^DR^ cells. Our results showed that vitexin treatment caused a reduced autophagic rate and autophagosome formation as observed by Cyto-ID and acridine orange (AO) staining in a concentration-dependent manner, respectively (Figure 4A and 4B). We then determined the effects of vitexin on the expression of proteins involved in the autophagy pathway. Western blot analysis showed that vitexin treatment markedly decreased the expression of autophagy maker proteins autophagy protein (ATG)5 and beclin-1 (BECN1), and suppressed the conversion of microtubule-associated protein light chain (LC3)-I to LC3-II (Figure 4C). These results indicated that vitexin inhibited autophagy in HCT-116^DR^ cells, which might be implicated in vitexin-mediated induction of apoptosis. To investigate whether this inhibitory effect is involved in vitexin-induced apoptosis, we used 3-methyladenine (3-MA), an autophagy inhibitor. Combination treatment with 3-MA and vitexin significantly decreased HCT-116^DR^ cell viability, as compared with HCT-116^DR^ cells treated with either 3-MA or vitexin (Figure 5A). Furthermore, co-treatment of HCT-116^DR^ cells with 3-MA and vitexin increased the cleavage of caspase-3 and caspase-9 and suppressed ATG5 and BECN1 expression, and LC3-I to LC3-II conversion, as compared to treatment with either 3-MA or vitexin (Figure 5B). These results indicated that autophagy inhibition contributed to vitexin-induced apoptosis.

### Vitexin inhibits tumor growth in a xenograft tumor model

**Figure 6:**
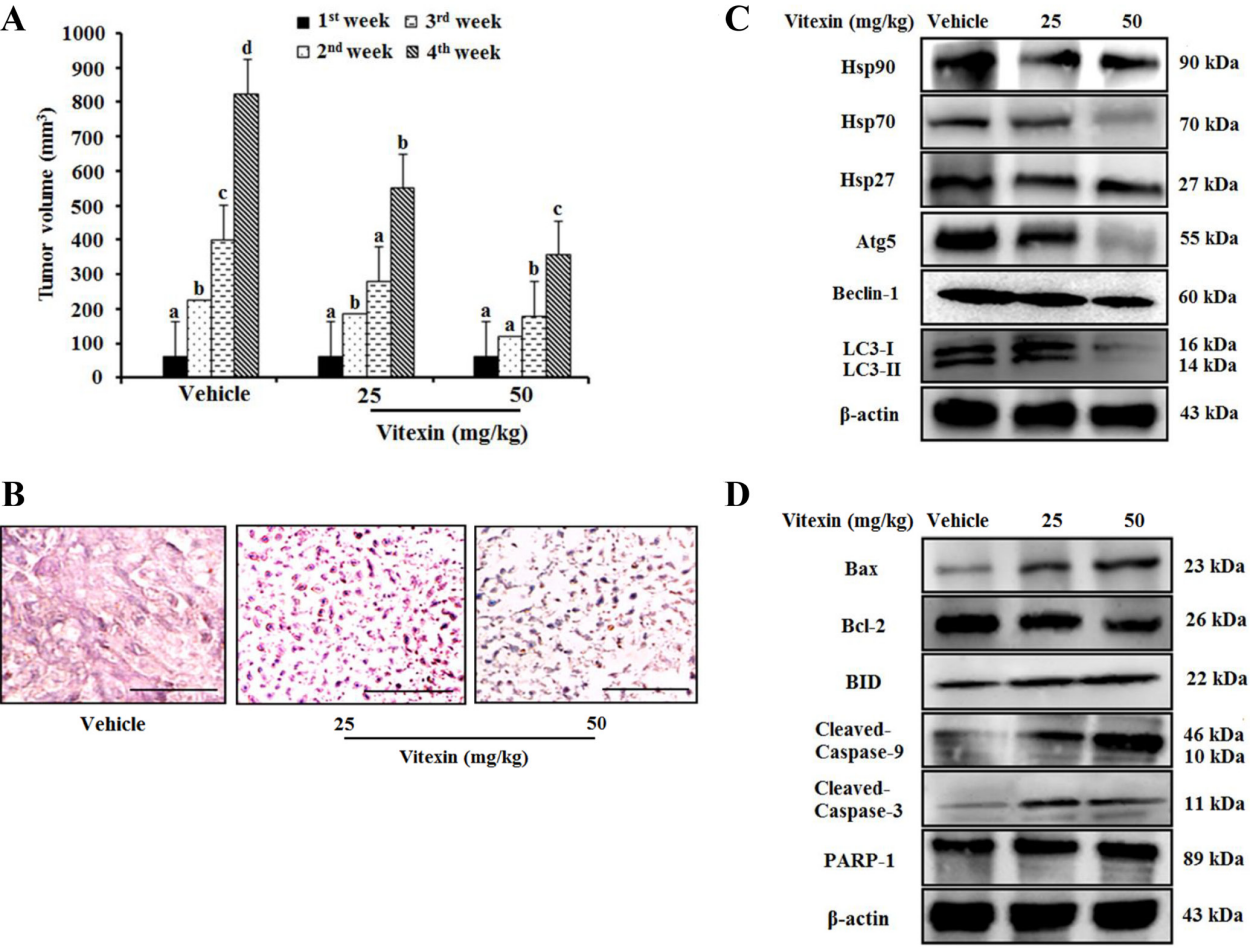
Vitexin treatment suppresses tumor growth in an HCT-116^DR^ tumor xenograft model 1 × 10^7^ HCT-116^DR^ cells were inoculated subcutaneously into the right flank region, and mice were monitored for tumor development. When tumors attained a size of 5 × 5 mm^3^, mice were received vehicle or 25 mg/kg or 50 mg/kg vitexin by oral administration for 3 days per week. (**A**) Tumor volume of mice were observed after initiation of treatment. (**B**) Representative images of tumor sections after H&E staining. HSPs and autophagy-related protein level (**C**), and apoptosis marker proteins (**D**) were analyzed by western blot. Data represent the mean ± SD of three independent experiments (*n* = 3). Values with different letters (a–d) denote significant differences from one another (*p* < 0.05).

Because vitexin inhibits cell viability *in vitro*, we then evaluated its antitumor effects in an animal model system. HCT-116^DR^ cells were injected subcutaneously into Balb/c nude mice, followed by oral administration of vitexin at doses of 25 mg/kg or 50 mg/kg. We observed that vitexin significantly reduced tumor size as compared with that observed in the vehicle-treated group (Figure 6A). Hematoxylin and eosin (H&E) staining demonstrated additional pathological changes associated with apoptosis observed in tumor tissues from the vitexin-treated group as compared with those from the vehicle group (Figure 6B). In the vehicle group, tumor cells had large, hyperchromatic and irregular nucleuses that were arranged in masses, with large nuclear-cytoplasmic areas (Figure 6B). In 25 mg/kg group, the tumor cells became smaller, apoptotic bodies were appeared but karyokinesis rarely was observed (Figure 6B). However, 50 mg/kg group induced extended regions of apoptotic and necrotic tumor tissue with a small number of the tumor cells experienced necrosis, and an increasing number of karyokinesises were visible (Figure 6B). Western blot analysis also revealed that vitexin treatment decreased the expression of HSP90 and HSP70, the anti-apoptotic protein Bcl-2, and the autophagy marker ATG5 as well as enhanced the expression of pro-apoptotic protein BAX and the cleavage of caspase-9 and caspase-3 (Figure 6C and 6D). These data indicated that the inhibitory effect of vitexin on tumor growth *in vivo* was attributable to autophagy inhibition and apoptosis induction.

### Influence of vitexin on zebrafish embryonic development

**Figure 7:**
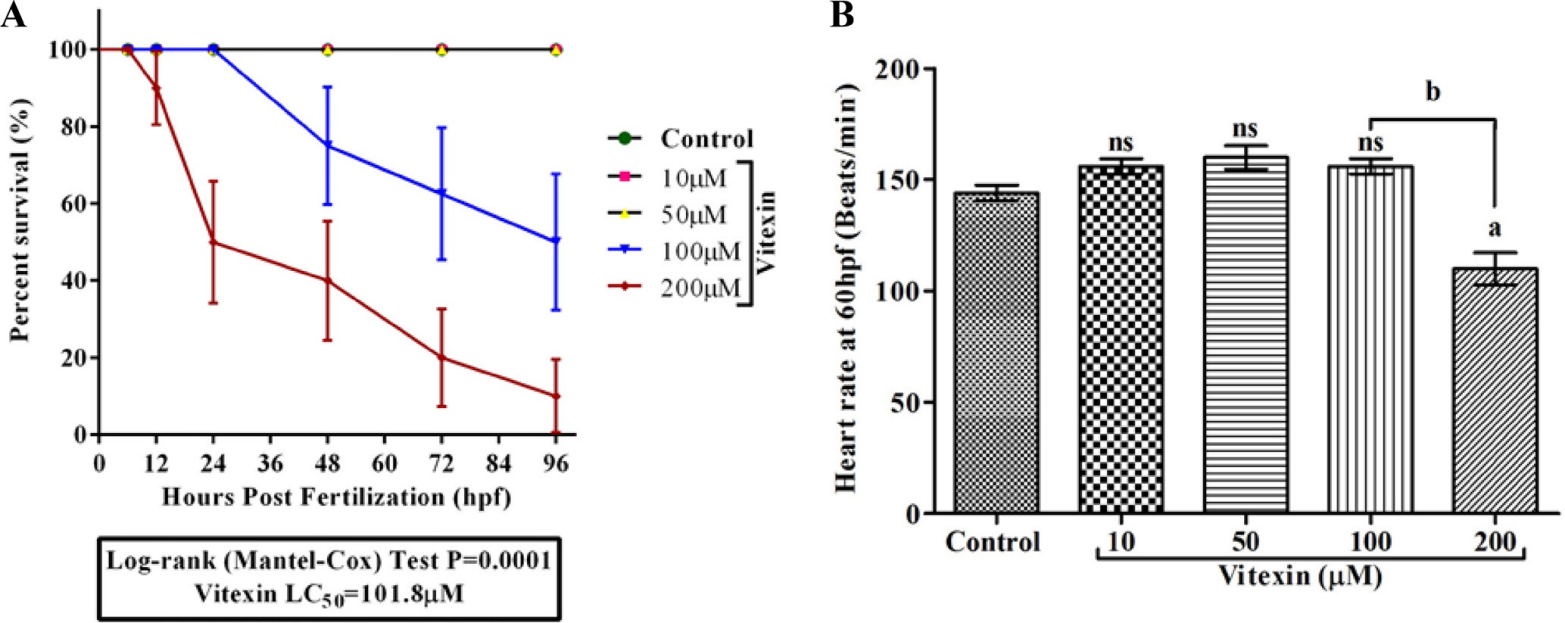
Effect of vitexin on embryonic development in zebrafish embryos for 96 hpf (**A**) Survival analysis using Kaplan-Meier test of zebrafish embryos after exposure with varying concentrations (10, 50, 100 and 200 µM) of vitexin. The analysis was performed using Mantel-Cox test (*P* = 0.0001). (**B**) The representative graph showed a heart rate of zebrafish embryo at 60 hpf. Values are expressed as mean ± S.E.M. (*n* = 3). Statistical variation from the control embryos are indicated by superscript letters (One-way ANOVA by Tukey’s test, Comparisons: ^a^*P* < 0.05 compared with control, ^ns^non-significant with control, ^b^*P* < 0.0001 vs 100 µM vitexin.

We further extended the toxicity study of vitexin in zebrafish embryonic development model. We initially monitored the growth patterns (Survival, hatching and morphological changes) to assess its toxicity. The embryos that were exposed to vitexin at concentrations ranging from 10, 50, 100 and 200 µM for 96 hpf showed a dose-dependent reduction in survival rate. As shown in the Figure 7A, vitexin at a 200 µM concentration significantly (*P* = 0.0001) caused alterations in survival rate (10 ± 9.4%) to that of 100 µM treated (50±17.6%). In contrast, 10 and 50 µM treated embryos did not elicit any significant sign of reduced survival rate when compared to control. The concentration required to reduce 50% of survival (LC_50_) was produced at 101.8 µM.

We next examined the influence of vitexin on zebrafish cardiac function (Figure 7B). At 60 hpf, control, 10 and 50 µM vitexin treated embryos exhibited normal morphology along with regular beats of 144 ± 3.4, 146 ± 2.3 and 147 ± 3.3 beats min^-1^ respectively, in the heart region. In contrast, 200 µM vitexin treated embryos displayed significantly (*P* < 0.001) reduced heart rate (110 ± 7.2 beats min^-1^) and its morphology was altered when compared to 100 µM (156 ± 3.4 beats min^-1^) vitexin exposed embryos. This result clear-cuts that cardiac functions were more susceptible to vitexin treatment at higher concentration.

**Figure 8:**
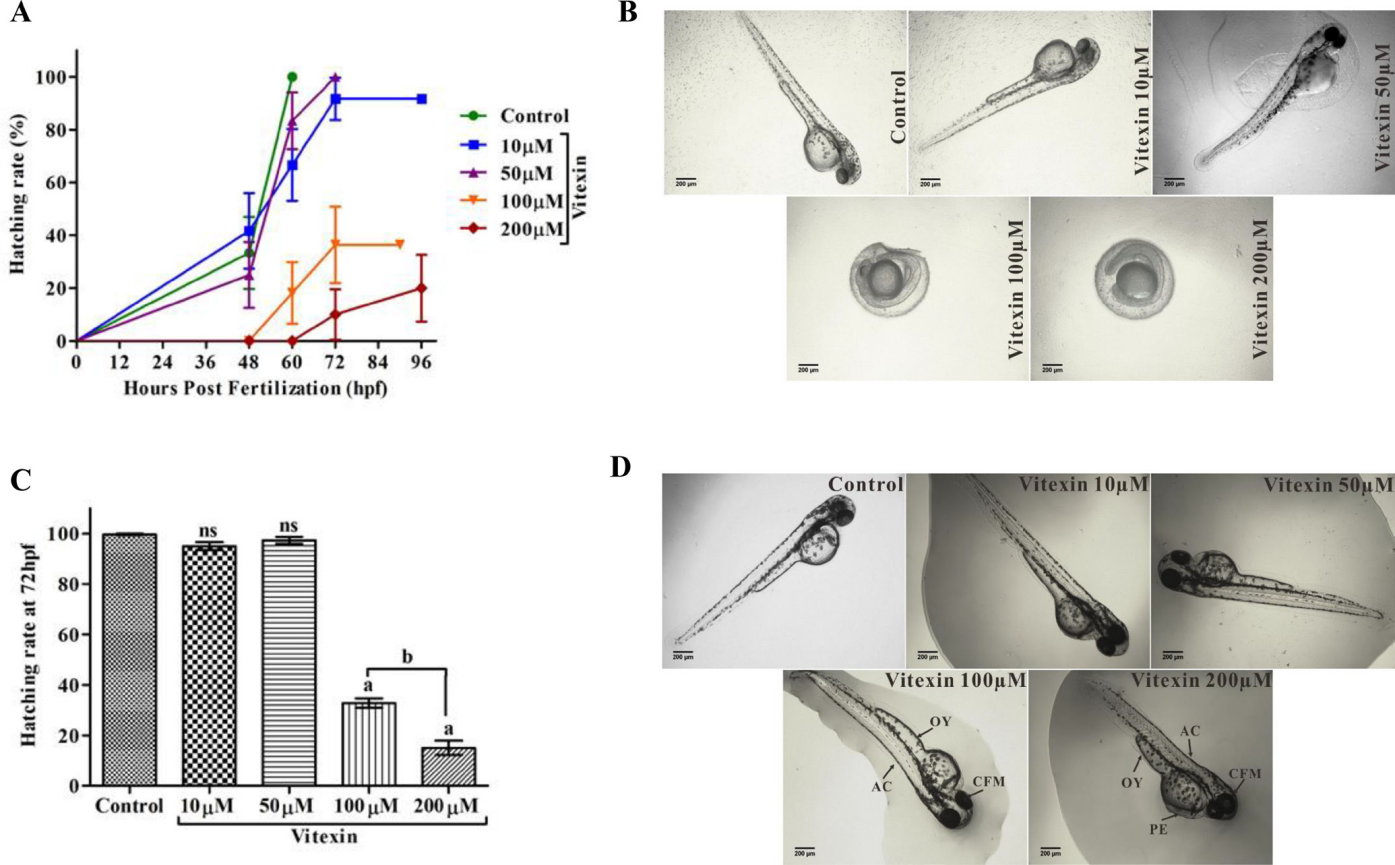
Influence of vitexin on zebrafish hatching and morphological changes (**A**) Overall hatching success of zebrafish after exposed with a dose-dependent concentration of vitexin (10, 50, 100 and 200 µM). (**B**) Delayed hatching morphology of zebrafish embryos at 72 hpf. (**C**) The graph represents the percentage of delayed hatching mechanism of zebrafish embryos induced by vitexin at (72 hpf). Data that are indicated by superscript letters are significantly different from the control embryos (One-way ANOVA by Tukey’s test, Comparisons: ^a^*P* < 0.0001 vs control embryos, ^ns^non-significant with control, ^b^*P* < 0.0001 vs 100 µM of vitexin. (**D**) Represents phenotypic changes of all experimental groups at 96 hpf. Arrow indicates severe malformations induced by vitexin at 100 and 200 µM concentrations, such as opaque yolk (OY), craniofacial malformation (CFM), and axial curvature (AC) when compared to control and other exposed doses of vitexin (Magnification ×4, Scale bar 200 µm).

When evaluating the hatching process (Figure 8A–8C), control and vitexin at 10 and 50 µM embryos did not show any delayed hatching process, whereas embryos exposed from 100 and 200 µM of vitexin showed significantly delayed hatching process in a concentration-dependent manner. Furthermore, treatment with vitexin > 100 µM also displayed a consistent and substantial pattern of morphological deformities including opaque yolk (OY), craniofacial malformation (CFM), and axial curvature (AC) when compared to < 100 µM doses of vitexin and control embryos (Figure 8D). Collectively, evidence from the developmental changes partly depicts that vitexin at above IC_50_ range causing adverse toxic to zebrafish embryos.

### Modulatory effect of vitexin on intracellular ROS and cell death events

**Figure 9:**
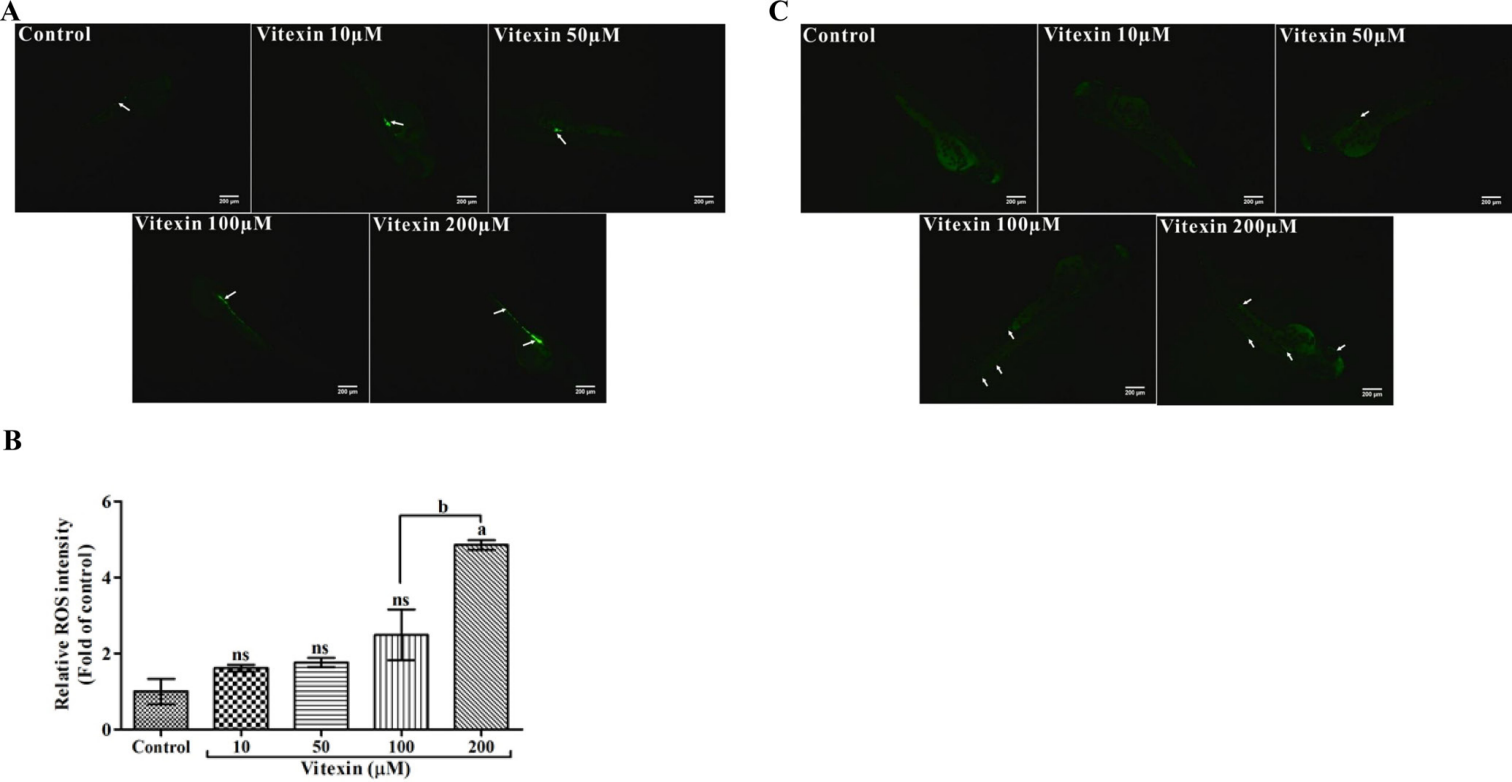
Effect of vitexin on the production of intracellular ROS levels in zebrafish larvae (**A**) The fluorescent probe DCFH-DA displays induced fluorescence intensities by vitexin at 200 µM in the intestinal region (white arrows and scale bar 200 µm). (**B**) Relative ROS intensities of all experimental larvae, the values are statistically significant (Tukey’s test), comparisons: ^a^*P* < 0.05, ^ns^non-significant with control larvae and ^b^*P* < 0.0001 vs 100 µM of vitexin. (**C**) Photomicrograph represents the acridine orange (AO) stained zebrafish larvae after treatment with vitexin in a dose-dependent manner (10, 50, 100 and 200 µM), Green fluorescent intensities evincing increased fluorescence of apoptotic bodies particularly in heart, tail and to some extent in eye regions (white arrows, scale bar 200 µm and magnification 4x).

The levels of intracellular ROS are considered as an index of cell death. To evaluate whether vitexin displaying pro-oxidant mediated cell death signatures in zebrafish embryos, we utilized fluorescent probe dichloro-dihydrofluorescein diacetate (DCFH-DA) a ROS indicator. As shown in the Figure 9A and 9B, control and <100 µM vitexin exposed embryos showed a lower intensity of ROS levels, which might be revealed as a host oxidative process. Whereas, 200 µM vitexin treated embryos showed significantly (*P* < 0.05) higher ROS intensities, particularly in the intestinal region (Figure 9A and 9B) when compared to 100 µM vitexin.

Enhanced ROS generation ultimately leads to cell death events; thus, we analyzed the apoptotic inducing capacity of vitexin using AO staining. As shown in the Figure 9C, control, 10 and 50 µM vitexin exposed larvae showed intact cells with reduced apoptotic bodies. In contrast, vitexin at 100 and 200 µM treated embryos could influence dose-dependent cell death as evidenced by the increased fluorescence intensity of AO at the heart, tail and eye regions. These results further strengthen our findings that cell death-inducing capacity above IC_50_ range might be due the pro-oxidant capacity of vitexin.

## DISCUSSION

ABC transporters bind to and hydrolyze a pair of ATP molecules and use this binding energy to efflux specific compounds across the membrane [18, 19]. Cancer cells can acquire chemoresistance through a number of mechanisms, including mutation or overexpression of the drug target, drug inactivation, or efflux of the drug from the cell. The acquisition of MDR represents a major obstacle for chemotherapeutic efficacy or several malignancies. One MDR-related-mechanism involves the surface accumulation of MDR1 (ABCB1/P-gp), which suppresses the influx and/or increases the efflux of anticancer drugs [20]. MDR1 is the first identified ABC transporter protein frequently overexpressed or amplified in a number of MDR human cancer cells, including those associated with renal, adrenocortical, colon and hepatocellular cancers [21]. Prolonged exposure to anticancer drugs can potentially lead to the selection of cells harboring elevated MDR1 expression and activity. In this study, we developed an MDR human colorectal carcinoma cell line (HCT-116^DR^) from parental HCT-116 cells. Indeed, HCT-116^DR^ cells showed the reduced cytotoxicity against various chemotherapeutic agents (Supplementary Figure 4) and the increased expression of MDR1 as compared to its parental cell line HCT-116 (Figure 1B).

HSF-1 was originally identified as a regulator of genes expressing HSP by binding to the heat shock element (HSE) located in their promoter regions. HSF-1 is localized to the cytoplasm as a monomer in unstressed cells; however, upon stimulation with stressors, HSF-1 oligomerizes and translocates to the nucleus, where it becomes transcriptionally active to promote the expression of HSPs and other cytoprotective target genes [22]. HSF-1 is often constitutively active in cancer cells and plays a multifaceted role in malignant transformation, including atypical expression of HSPs. Several reports show that HSF-1 regulates MDR-1 expression at the transcriptional level and confers MDR of cancer cells against various chemotherapeutic agents [23, 24]. Additionally, HSEs have been identified in the *MDR1* promotor region, and stress inducers, such as heat-shock and arsenite, induce the expression of *MDR1,* as well as *HSP* genes [25, 26]. In agreement with these findings, our results showed that vitexin inhibited HSF-1 nuclear translocation and resulted in the decreased expression of MDR1, HSP90 and HSP70 (Figure 2D and Figure 3A), suggesting that vitexin-mediated reductions of MDR1 levels might be attributable to suppression of HSF-1 nuclear translocation.

Vitexin exhibits anti-tumor efficacy against various human cancers, including ovarian, prostate, and breast cancers by inhibiting the growth and promoting apoptosis of cancer cells [8, 9]. Resistance might result from failure to initiate apoptosis activated in response to drug treatment. As the evolutionarily conserved form of active cell death, apoptosis involves cell-mediated self-destruction in response to a number of stimuli, including growth-factor deprivation and antitumor drugs. Apoptosis encompasses a broad range of morphological processes that are accompanied by membrane blebbing, chromosomal DNA fragmentation, chromatin condensation, and cell shrinkage [27]. Caspases are activated in a cascade-like manner with pro-apoptotic signals, such as chemotherapeutic agents, activating initiator caspases (caspase-8 and caspase-9) to initiate the proteolytic cascade. These initiator caspases activate effector caspases (caspase-3 and caspase-7), which lead to the biochemical and morphological changes that are characteristic of apoptosis [28]. The Bcl-2 family includes the most well-characterized regulators of apoptosis, such as the anti-apoptotic members, Bcl-2 and Bcl-X_L_, and the pro-apoptotic members, BAX, Bcl-2-homologous antagonist/killer (BAK), and BID [29]. The present study showed that vitexin treatment increased levels of pro-apoptotic Bcl-2 members (BAX and BID) and promoted the cleavage of caspase-3, resulting in apoptosis induction. Therefore, vitexin-induced apoptosis contributes to the suppression of drug-resistant colon cancer cell line, HCT-116^DR^, which may be a promising chemotherapeutic agent to overcome drug-resistance in cancer cells.

Autophagy is a highly conserved cellular process, in which cytoplasmic components are engulfed in vesicles and delivered to lysosomes for degradation [30]. Therefore, it plays an essential role in both the maintenance of cell homeostasis and the recycling of damaged organelles and misfolded proteins. Autophagosome formation depends on the processing of various marker, such as up-regulation of ATG5 and ATG7, and conversion of LC3-I to LC3-II [31]. Autophagy is positively or negatively interconnected with tumor development and implicated in the treatment of a variety of diseases, including cancer, with previous study reporting that various natural products represent effective chemotherapeutic agents through their capacity to inhibit autophagy [32]. Our *in vitro* and *in vivo* studies showed that vitexin suppressed autophagosome formation and decreased the expression of autophagy marker proteins (ATG5 and BECN1) and the conversion of LC3-I to LC3-II in HCT-116^DR^ cells (Figure 4 and 6). Furthermore, co-treatment with the autophagy inhibitor 3-MA and vitexin markedly decreased the viability of HCT-116^DR^ cells and increased the activation of caspase-3 and caspase-9 (Figure 5).

The biochemical properties of vitexin are well-documented due to their strong antioxidant [33], anticancer [34] and neuroprotective [35] properties. However, the possible side-effects of vitexin must be studied for the further usage of vitexin in clinical setting. Using zebrafish as a model organism, we have investigated first here the effect of vitexin on early life-stages of zebrafish development by assessing the general observations (Survival, hatching and heart rate), reactive oxygen species levels (ROS) and apoptotic makers for 96hpf. Our evidence showed that exposure to low concentration of vitexin (< 100 μM) could not affect the survival, cardiac function, hatching and/or obvious malformation for 96 hpf (Figure 7 and Figure 8). Controversially, vitexin at higher doses (≥ 100 μM) exhibited significantly reduced survival rate, significantly affected the heart rate, hatching process along with severe phenotypic deformities in zebrafish embryos (Figure 7 and Figure 8). It seems that vitexin produced lowest oxidative stress-driven toxic effect on zebrafish embryos. Our results are in line with other reports addressing the lower toxic impact of phenol and flavonoids on zebrafish embryo development [36].

Several reports stressed those plant-derived or synthetic compound exhibited prooxidant or cytotoxic properties under certain conditions (Presence of oxygen levels, metal interation and alkaline pH) [37]. A wealth of reports stressed that ROS production involves in the regulation of apoptotic and other signaling cascades under pathological conditions [38]. DCFH-DA and AO staining showed that only limited levels of ROS and apoptotic bodies were observed up to 50 μM vitexin zebrafish (Figure 9A and 9B). In contrast, the higher levels of ROS production and signature of apoptotic bodies (Figure 9A and 9B) were enhanced by vitexin treatment (200 μM). These finding corroborated the ROS-mediated apoptotic effect to vitexin above lethal concentration (≥ 100 μM) and below lethal concentration (≤ 100 μM) does not exhibit any severe side effects. Therefore, our findings suggested that vitexin treatment inhibited the pro-survival effects of autophagy, thereby exhibiting potent anti-tumor by promoting apoptosis induction in MDR HCT-116^DR^ cells. Collectively, our finding provide insight into the therapeutic potential of vitexin for the treatment of chemoresistant colorectal cancer.

## MATERIALS AND METHODS

### Chemicals

Vitexin, acridine orange (AO), Tricaine, 5-fluorouracil (5-FU), cisplatin (CIS), docetaxel (DOC), vincristine (VIN), 3-(4,5-dimethyl-2-thiazolyl)-2,5-diphenyl-2H-tetrazolium bromide (MTT), Hoechst 33342, 3-methyladenine (3-MA), dimethylsulfoxide (DMSO), dichloro-dihydrofluorescein diacetate (DCFH-DA) and other chemicals, unless stated otherwise, were purchased from Sigma-Aldrich (St. Louis, MO, USA). β-actin, MDR-1, HSF-1, lamin B, HSP70, HSP27, BID, BAX, cytochrome C, caspase-3, caspase-9, and Bcl-2 antibodies were obtained from Santa Cruz Biotechnology, Inc. (Santa Cruz, CA, USA). HSP90 antibody was purchased from Enzo Life Science (Farmingdale, NY, USA). BECN-1 and ATG5 were purchased from Cell Signaling Technology (Beverly, MA, USA). LC3 was obtained from Abcam (Cambridge, MA, USA). Horseradish peroxidase (HRP)-conjugated secondary antibodies for western blot analysis were purchased from Santa Cruz Biotechnology.

### Cell culture and establishment of the MDR HCT-116 cell line

The human colorectal carcinoma cell line HCT-116 (ATCC, Rockville, MD) was maintained at 37°C (95% air, 5% CO_2_) in RPMI 1640 medium (Gibco BRL, Gaithersburg, MD, USA) supplemented with 10% fetal bovine serum (FBS), 25mM HEPES buffer, and 1% Pen-Strep cocktail (Gibco BRL). The process for selection and establishment of drug-resistant HCT-116 cell lines was established in our department. Briefly, HCT-116 cells at low density were seeded into a 25 cm^2^ flask and treated with 0.2 nM docetaxel, vincristine, and 0.2 µM cisplatin and 5-fluorouracil until the surviving cells grew into an obvious colony. These cells were exposed three times in a 3-day period over the course of 3–6-weeks, allowing for growth recovery between cycles. After completion of three cycles of drug treatment, the doses were doubled, and the procedure was repeated until treatment with the final drug concentrations were achieved. All cell lines were maintained as a monolayer in complete medium. A selected colony was amplified in the presence of all drugs until confluence before the drug dose was increased in multiples of two for the next round of selection. The MDR sub-line maintained at 10 nM docetaxel, vincristine, and 10 µM cisplatin and 5-FU was denoted as HCT-116^DR^. Experiments related with other cell lines and zebrafish embryo model are included in Supplementary materials and methods.

### MTT and LDH assays

Following vitexin treatment for 24 h, cell cytotoxicity was assessed by measuring the optical density at 540 nm with a microplate reader (Bio-Tek Instruments, Winooski, VT, USA) 4 h after the addition of MTT working solution (5 mg/mL). Plasma-membrane integrity was assessed based on lactate dehydrogenase (LDH) leakage into the culture medium from cells using an LDH assay kit (Sigma-Aldrich). LDH leakage was determined by measuring the optical density at 490 nm and 690 nm.

### Detection and quantification of acidic vesicular organelle (AVO) formation

The presence of AVOs was assessed by AO staining. Briefly, compound-treated cells were washed with PBS, followed by staining with 1 μg/mL AO diluted in PBS containing 5% FBS for 30 min at 37°C. Cells were washed with PBS and then observed at 488 nm under a fluorescence microscope equipped with filters. For AVO quantification, AO–stained cells were harvested, washed twice with PBS, resuspended in PBS containing 5% FBS, and then analyzed by fluorescence microscopy.

### Western blot analysis

Cell lysates (whole-cell, cytoplasmic, and nuclear) were separated on 10% or 15% polyacrylamide gels and transferred onto a polyvinylidene difluoridemembrane. The blot was subsequently incubated with 5% non-fat milk in PBS for 1 h, probed with antibody against a specific protein for 2 h at 37°C or overnight at 4°C, and then probed with an appropriate HRP-conjugated secondary antibody for 1 h at room temperature. Washing between incubations was conducted with wash buffer three times, and after the final washing, signals were developed using the ECL detection system.

### Rh-123 assay for P-gp activity

P-gp-related efflux activity was evaluated by the ability of the cells to detect the fluorescent compound Rh-123. Cells were seeded at 1 × 10^5^ cells/well in 6-well plates. After cell treatment with vitexin (10, 25, and 50 μM) for 24 h, the medium was replaced with fresh medium containing Rh123 (final concentration; 1 μM) and incubated for 30 min at 37 °C. After incubation, cells were washed twice with cold PBS and re-suspended in PBS for further detection of Rh-123 fluorescence (Nikon Eclipse TS100 Epi-fluorescence microscope, Japan). The mean fluorescence value was converted to the percentage of that observed in the control.

### ATPase assay

For active drug efflux, P-gp ATPase activity needs to catalyze the decomposition of ATP into ADP and free phosphate. HCT-116 ^DR^ cells were cultured and treated with vitexin at various concentrations for 24 h. The reaction was initiated by addition of 5 mM ATP, followed by incubation at 37°C for 20 min. The reaction was terminated by addition of 5% sodium dodecyl sulfate solution, and free phosphate liberated by the enzyme and resulting in a colorimetric product, was measured to determine its proportionality to the enzyme activity present (Sigma-Aldrich, St. Louis, USA).

### Comet assay

To evaluate DNA damage, frosted glass microscopic slides were precoated with 1% normal melting-point agarose. HCT-116^DR^ cells were harvested after treatment and then suspended in 70 µL of 1% low-melting-point agarose in PBS (pH 7.4) at 37°C. Cells were immediately pipetted onto precoated frosted slides, and the agarose gels were allowed to set up for 10 min on ice. Cells were lysed by placing the slides in a Coplin jar containing 2.5 M NaCl, 0.1 M Na_2_ EDTA, 10 mM Tris, 10% DMSO and 1% Triton X-100 (pH 10) at 4°C for at least 1 h. Slides were then immersed in electrophoresis solution (0.3 M NaOH and 1 mM Na_2_ EDTA, pH > 13) for 30 min, and electrophoresis was performed at 1.3V/cm for 20 min in the same solution, followed by washing twice with neutralization buffer (0.4 M Tris, pH 7.5) for 20 min. Comets were fixed by immersing the slides in 70% ethanol for 15min and in absolute ethanol for a further 15min before placing them on the bench to dry overnight. Comets were stained with SYBR green at the dilution recommended by the manufacturer (Sigma-Aldrich). One hundred comets on each slide were quantified with computerized image analysis using Komet 3.0 (Kinetic Imaging Ltd, Liverpool, UK) according to the relative intensity of fluorescence in the tail and results were grouped.

### Detection of nuclear condensation by Hoechst staining

After treatment, cells were washed twice with PBS and fixed with 4% formaldehyde for 30 min. The fixed cells were stained with Hoechst 33342 dye (1 μg/mL) for 15 min in the dark and washed with PBS [25], and fluorescent micrographs were obtained using a Nikon Eclipse TS100 Epi-fluorescence microscope (Nikon).

### Flow cytometry analysis

Apoptosis induction and AVO development were quantified using an *in situ* Cell Death Detection Kit (Roche Applied Sciences, Basel, Switzerland) and Cyto-ID™ Autophagy Detection Kit (Enzo LifeSciences), respectively, and analyzed by flow cytometry. Cells were stained for 30 min at 37°C and then harvested, after which green (510–530 nm) fluorescence emission from 1 × 10^4^ cells illuminated with blue (488nm) excitation light was measured using a fluorescent activated cell sorter. A total of 50,000 events were collected, and data analysis was performed using Kaluza flow cytometry software (Beckman Coulter, Fullerton, CA, USA).

### Measurement of single-stranded DNA

Amounts of single-stranded DNA in cells were determined using ApoStrand ELISA apoptosis detection kit (Enzo Life Sciences). DNA in apoptotic cells is sensitive to formamide denaturation, and denatured DNA was detected using a monoclonal antibody against single-stranded DNA. 1 × 10^5^ cells were cultured in 96-well plates and subjected to vitexin treatment. The assay was performed according to manufacturer instructions, and absorbance was measured at 405 nm with an ELISA reader (Bio-Tek Instruments).

### Athymic nude mice xenograft study

Female BALB/c pathogen-free athymic nude mice, (4-weeks-old; body weight 20–22 g) were purchased from Orient Bio, Inc. (Seoul, South Korea). Mice were housed in a sterile temperature controlled-room on a 12 h:12 h light:dark schedule with a standard rodent chow diet and water *ad libitum*. The guidelines for animal care and handling were approved by the Institutional Animal Care and Use Committee of Daegu University (Kyoungbook, South Korea). For engraftment, 1x10^7^ HCT-116^DR^ cells were inoculated subcutaneously into the right flank region, and mice were monitored for tumor development. When tumors attained a size of 5 x 5 mm^3^, mice were randomly assigned to four groups (*n = 5*/group) and received vehicle or 25 mg/kg or 50 mg/kg vitexin by oral administration for 3 days per week. Tumor volumes were monitored for the duration of the experiment and estimated using the formula [(W)^2^ x L]/2, where W represents the width (shortest tumor diameter) and L represents the length (longest tumor diameter). Tumors were dissected and stored in liquid nitrogen or fixed in formalin for further analysis.

### Hematoxylin and Eosin staining

Formalin-fixed, paraffin-embedded tumor specimens were sectioned (4 µM), and after deparaffinization and hydration, the slides were washed with distilled water and stained with hematoxylin for 5 min to 10 min. Excess amounts of stain were removed with 1% HCl-EtOH solution, followed by washing with ammonia water and distilled water. Background counterstaining was performed with eosin, followed by ethanol and xylene washes, and slides were then analyzed under a light microscope (400x).

### Data analysis and statistical procedures

Statistical analysis was performed using SPSS software v22.0 for Windows (IBM Corp., Armonk, NY, USA). Results are expressed as the mean ± standard deviation (SD) of three independent experiments. Data were subjected to one-way analysis of variance, and the significant differences between samples were calculated using Duncan’s multiple range test. Differences were considered statistically significant at *p* < 0.05.

## SUPPLEMENTARY MATERIALS FIGURES


