# Supplementary material for: Vitexin induces apoptosis by suppressing autophagy in multi-drug resistant colorectal cancer cells
Source: Oncotarget. 2017 Dec 4;9(3):3278–91. doi: 10.18632/oncotarget.22890 (PMC5790463; doi:10.18632/oncotarget.22890)
Supplement: Supplementary file 1 [file oncotarget-09-3278-s001.pdf]

Vitexin induces apoptosis by suppressing autophagy in multi-drug resistant colorectal cancer cells

SUPPLEMENTARY MATERIALS

MATERIALS AND METHODS

Cell culture and MTT assay

The human colorectal carcinoma cell line HCT-116, Km12C and HT29, human lung epithelial carcinoma cell line A549 and docetaxel resistant A549 Dox (ATCC, Rockville, MD) were maintained at 37°C (95% air, 5% CO_2) in RPMI 1640 medium (Gibco BRL, Gaithersburg, MD, USA) supplemented with 10% fetal bovine serum (FBS), 25mM HEPES buffer, and 1% Pen-Strep cocktail (Gibco BRL). The human hepatocellular carcinoma cell line HepG2, cisplatin resistant HepG2 Cis and human cervical cancer cell line HeLa were cultured in DMEM (Sigma-Aldrich (St. Louis, MO, USA), supplemented with 10% fetal bovine serum (FBS) and 1% Pen-Strep cocktail (Gibco BRL). Following vitexin treatment (25–100 µM) for 24 h, cell cytotoxicity was assessed by measuring the optical density at 540 nm with a microplate reader (Bio-Tek Instruments, Winooski, VT, USA) 4 h after the addition of MTT working solution (5 mg/mL).

Zebrafish and maintenance

Zebrafish (*Danio rerio*) were purchased from the local aquarium and maintained at 27 ± 1°C in a glass tank equipped with water aeration system under 14 h light/10 h dark cycle (Westerfield, 1995). For embryo collection, 2:1 ratio of female and male zebrafish were allowed in a steel meshed tank and the spawning was induced by the onset of light illumination in morning. The fertilized eggs were collected and then maintained in embryo (E3) medium (0.2 mM $Ca(NO_3)_2$, 0.13 mM $MgSO_4$, 19.3 mM NaCl, 0.23 mM KCl, and 1.67 mM HEPES) for toxicity analysis. For experiment analysis, embryos were anesthetized with tricaine (4 mg/mL) with the dilution of 1:100.

Preparation of vitexin

Vitexin was solubilized in DMSO and was exposed at the dose of 10, 50, 100 and 200 µM in E3 medium for 96 hpf.

Exposure of zebrafish embryos to vitexin

To investigate the lethal impact of vitexin on zebrafish developmental stages, embryos at 2 hpf (hours post fertilization) were randomly sorted into five groups ($n = 20$) in a six well plates. Embryos in group 1 served as control and received 0.1% of DMSO for 96hpf. Embryos in groups 2 to 5 were incubated with dose-dependent concentrations of vitexin (10, 50, 100 and 200 µM for 96hpf. Developmental changes including survival, hatching, heartbeat and morphological changes were recorded at specific time intervals (24, 48, 72 and 96 hpf) under a Nikon Eclipse TS200 microscope.

Analysis of ROS and apoptotic signatures

To evaluate the dose-dependent ROS and cell death inducing capacities by vitexin, 2 days old larvae of all experimental groups were incubated with acridine orange (7.5 µM in E3 medium) for 30 min in dark. After incubation, the larvae were washed with excess medium and then visualized under a fluorescent microscope (Excitation wavelength-488 nm, Nikon Eclipse TS100 Epi-fluorescence microscope, Japan). Similarly, for ROS analysis, 2 days old post treated larvae were incubated with a ROS indicator- dichloro-dihydrofluorescein diacetate (DCFH-DA; 5 µM) for 30 min in dark. Finally, embryos were washed thrice with E3 medium and then changes in ROS intensities were photographed using a fluorescent microscope (488 nm excitation). The representative intensities were quantified using ImageJ Software.

Statistical analysis

All the data was evaluated using GraphPad Prism Software (Version 5.0, San Diego, California, USA). The values are expressed as mean ± S.E.M. ($n = 3$). The lethal concentration (LC_{50}) was performed using non-regression curve fit and the survival rate was analysed using Kaplan-Meier test. Hypothesis testing included one-way analysis of variance (ANOVA) was analyzed by Tukey's test. Values of $P < 0.05$ was considered to represent as statistical significance.

REFERENCES

Westerfield M. The Zebrafish Book, a Guide for the Laboratory Use of Zebrafish (*Danio rerio*), 3rd ed. University of Oregon Press, Eugene, OR, USA. 1995.

A

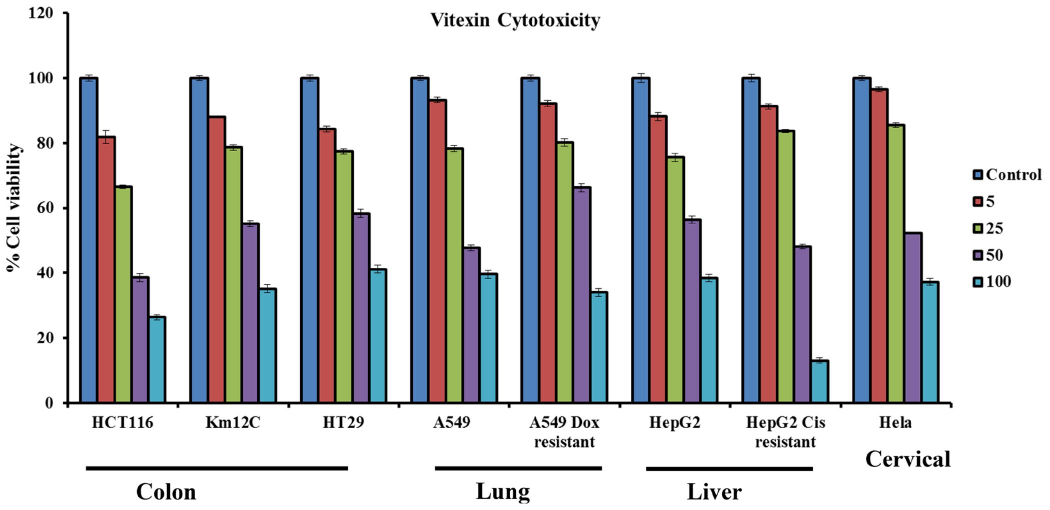

B

Cells	Ic50 (µM)
HCT116	35.65
KM12C	52.35
HT29	55.65
A549	44.58
A549 Dox	60.31
HepG2	44.32
HepG2 Cis	46.31
Hela	48.55

Supplementary Figure 1: Effect of vitexin on cell viability in various cell lines. (**A**) Different cell lines were treated with the indicated concentration of vitexin for 24 h, and cell viability were determined by MTT assay [Cis = Cisplatin; Dox = Docetaxel]. (**B**) IC50 value of vitexin for different cell line was calculated. Data represents the mean ± SD of three independent experiments (n = 3).

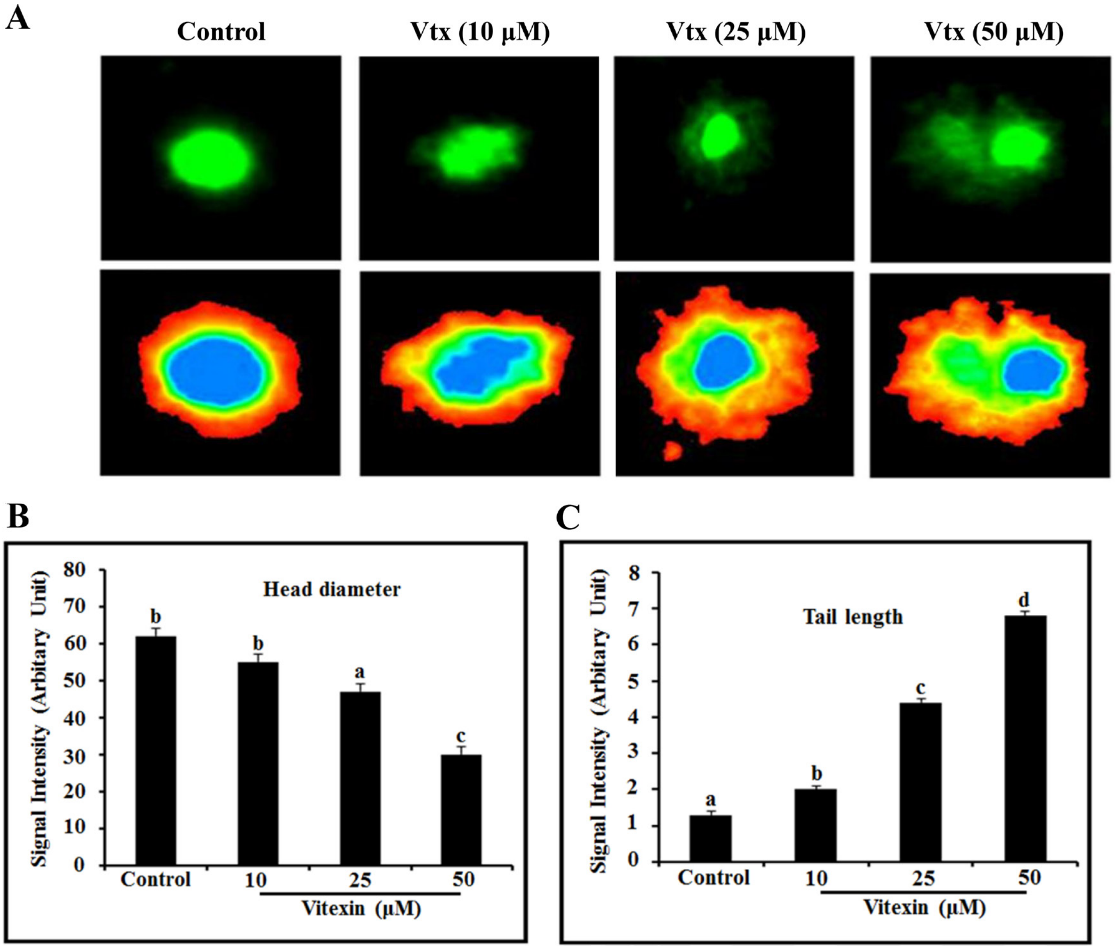

Supplementary Figure 2: Vitexin induces DNA damage in HCT-116DR cells. (**A**) After 24 h of incubation, HCT-116DR cells were subjected to comet assay. The presence of comet tails (halo effect) indicates the presence of fragmented DNA. The percentage of DNA in tail has been increased after incubation with vitexin. Quantification of (**B**) head diameter (**C**) tail length was calculated after comet assay. Nuclei with DNA damage (with comet) showed reduced head diameter and increased tail length. The data represent mean ± SD of three independent experiments, n = 3. Values with different letters (a-d) differ significantly from each other ($p < 0.05$).

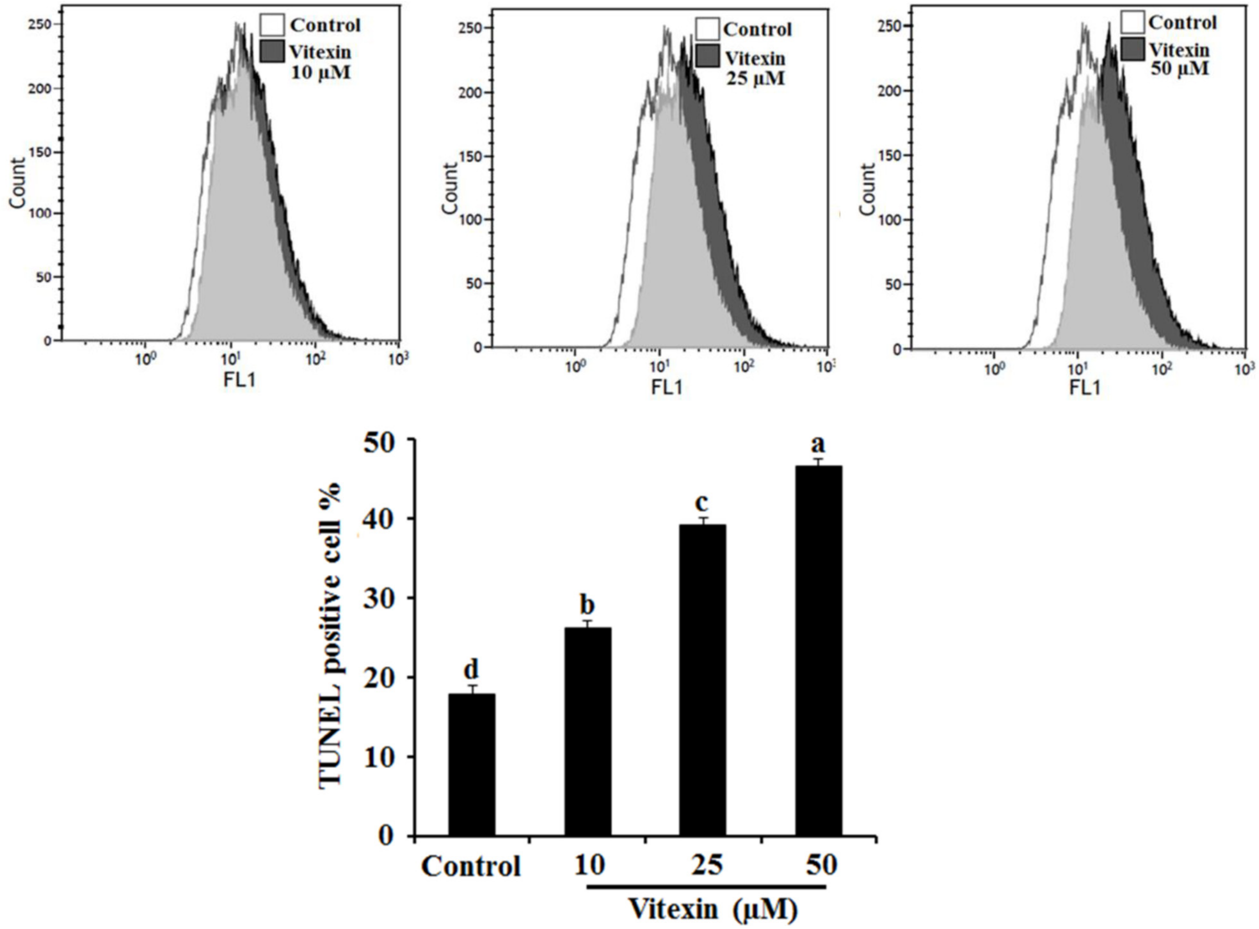

Supplementary Figure 3: Vitexin induces apoptosis-mediated cell death. Cell apoptosis was analyzed by TUNEL assay flow cytometry. Representative graph of TUNEL positive cell percentage determined by flow cytometry after treatment with vitexin for 24 h. The data represent mean ± SD of three independent experiments, $n = 3$. Values with different letters (a-d) differ significantly from each other ($p < 0.05$).

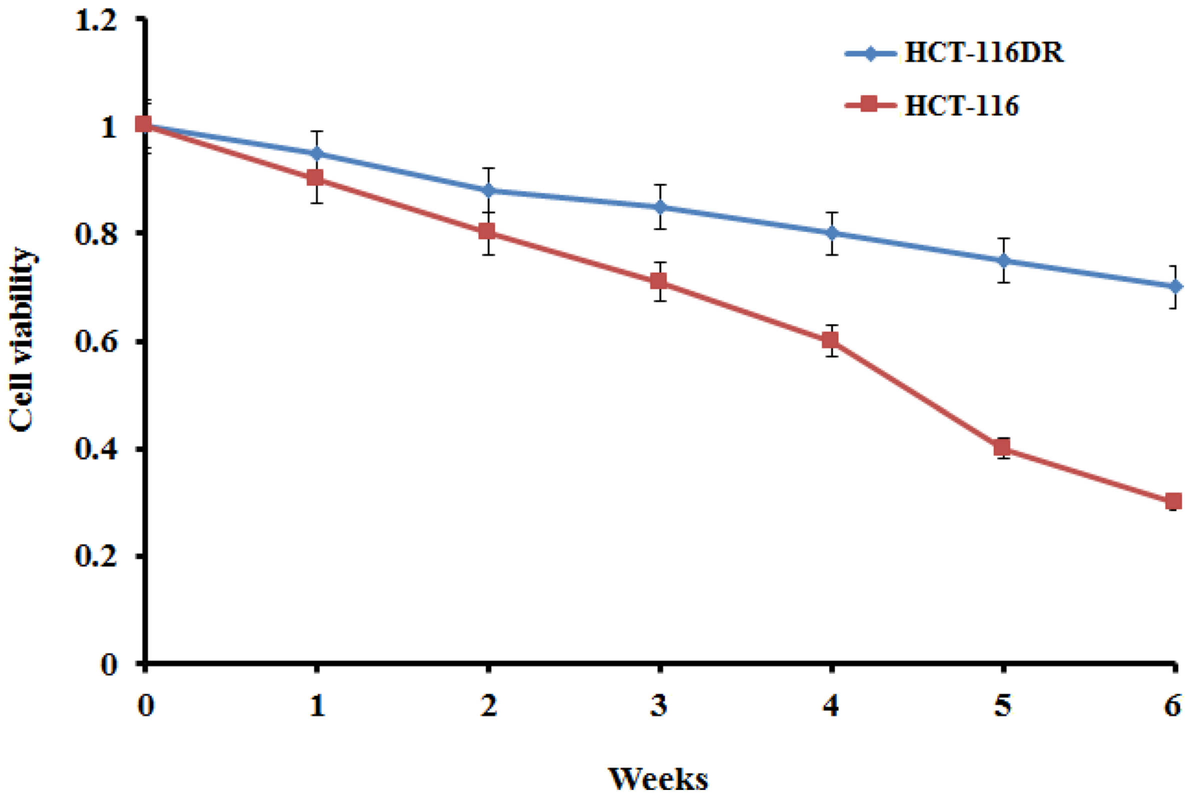

Supplementary Figure 4: HCT-116^{DR} cell line was established. HCT-116 cells were treated with 0.2 nM docetaxel, vincristine, and 0.2 µM cisplatin and 5-fluorouracil for three times in a 3-day period over the course of 3–6-weeks, allowing for growth recovery between cycles. Cell viability was assessed by MTT assay. The MDR sub-line maintained at 10 nM docetaxel, vincristine, and 10 µM cisplatin and 5-FU was denoted as HCT-116DR. Data represent the mean ± SD of three independent experiments (*n* = 3).
